# Analysis of global DNA methylation changes in primary human fibroblasts in the early phase following X-ray irradiation

**DOI:** 10.1371/journal.pone.0177442

**Published:** 2017-05-10

**Authors:** Anna Maierhofer, Julia Flunkert, Marcus Dittrich, Tobias Müller, Detlev Schindler, Indrajit Nanda, Thomas Haaf

**Affiliations:** 1 Institute of Human Genetics, Julius Maximilians University, Würzburg, Germany; 2 Department of Bioinformatics, Julius Maximilians University, Würzburg, Germany; ENEA Centro Ricerche Casaccia, ITALY

## Abstract

Epigenetic alterations may contribute to the generation of cancer cells in a multi-step process of tumorigenesis following irradiation of normal body cells. Primary human fibroblasts with intact cell cycle checkpoints were used as a model to test whether X-ray irradiation with 2 and 4 Gray induces direct epigenetic effects (within the first cell cycle) in the exposed cells. ELISA-based fluorometric assays were consistent with slightly reduced global DNA methylation and hydroxymethylation, however the observed between-group differences were usually not significant. Similarly, bisulfite pyrosequencing of interspersed LINE-1 repeats and centromeric α-satellite DNA did not detect significant methylation differences between irradiated and non-irradiated cultures. Methylation of interspersed ALU repeats appeared to be slightly increased (one percentage point; p = 0.01) at 6 h after irradiation with 4 Gy. Single-cell analysis showed comparable variations in repeat methylation among individual cells in both irradiated and control cultures. Radiation-induced changes in global repeat methylation, if any, were much smaller than methylation variation between different fibroblast strains. Interestingly, α-satellite DNA methylation positively correlated with gestational age. Finally, 450K methylation arrays mainly targeting genes and CpG islands were used for global DNA methylation analysis. There were no detectable methylation differences in genic (promoter, 5' UTR, first exon, gene body, 3' UTR) and intergenic regions between irradiated and control fibroblast cultures. Although we cannot exclude minor effects, i.e. on individual CpG sites, collectively our data suggest that global DNA methylation remains rather stable in irradiated normal body cells in the early phase of DNA damage response.

## Introduction

Radiation therapy is a highly effective form of cancer treatment. Depending on the type of cancer and the personalized therapies prescribed to individual patients, it is either used alone or usually in combination with chemotherapy or other treatments. However, therapeutic irradiation does not only affect the intended neoplastic targets but also normal body cells in surrounding tissues. Ionizing radiation induces DNA damage either directly in exposed cells or indirectly (delayed) in cells several generations after exposure. Predominant DNA lesions are base damages, single- and double-strand breaks (DSBs), and DNA-protein crosslinks [1,2]. Accumulating evidence suggests that irradiation not only induces DNA damage and genome instability but also epigenetic alterations, in particular DNA methylation changes [3–6]. So far the main focus of epigenetic studies has been DNA methylation alterations several population doublings after irradiation, when radiation-induced genome instability may occur in cells that were not directly exposed [7–9]. In contrast, our study is focused on direct epigenetic effects in cells within the first cell cycle after irradiation.

Epigenetic processes are crucial for maintaining cellular homeostasis and their dysregulation can lead to malignant transformation. DNA methylation is the most thoroughly studied epigenetic modification and occurs at the carbon 5’ atom of cytosine, mainly in the context of cytosine-phosphate-guanine (CpG) dinucleotides. CpG islands (CGIs) are 500–2,000 bp segments of high CpG density in the vertebrate genome. CGIs occur in the promoter and/or first exon of most mammalian genes. Methylation of these cis-regulatory CGIs during development, differentiation, and disease processes leads to an inactive chromatin structure and gene silencing. In contrast, gene body methylation is usually associated with active genes [10,11]. Apart from CGIs, methylated CpGs are enriched in repetitive DNA elements to prevent retrotransposition activity and maintain genome integrity [12,13]. Compared to methylation, hydroxymethylation is a relatively rare DNA modification that is found in different mammalian tissues with the highest concentration in brain [14–15]. For active (replication-independent) DNA demethylation, 5-methylcytosine (5-mC) is first converted to 5-hydroxymethylcytosine (5-hmC), and then to 5-formylcytosine and 5-caroboxylcytosine [16,17]. This reaction is catalyzed by the ten-eleven translocation (TET) family of enzymes [18] and may be involved in epigenetic gene regulation [19,20].

Human cancer cells show dramatic alterations in DNA methylation and hydroxymethylation compared to normal human cells [21,22]. Hypermethylation of CGIs in promotor regions leads to inactivation of tumor suppressor genes, whereas global hypomethylation of repeat elements leads to reactivation of retrotransposons. Both processes interfere with genomic stability [23]. Global hydroxymethylation is also decreased in multiple types of cancers [24,25]. Studies on the effects of ionizing radiation on DNA methylation have yielded conflicting results. Antwith *et al*. [3] analyzed DNA methylation changes in breast cancer cells over a period of 72 h after radiation treatment using 450K methylation arrays. Differentially methylated genes were enriched in gene ontology categories relating to radiation response pathways. Another 450K array study [4] on irradiated colon cancer cells revealed global DNA methylation changes and reduced methylation at specific gene loci. In contrast, Lahtz *et al*. [26] did not find significant DNA methylation changes in normal human fibroblasts and bronchial epithelial cells 7 days after low-dose (0.1–1 Gy) γ-irradiation, using a methylated CGI recovery assay combined with microarrays.

In our study, we analyzed global DNA methylation and hydroxymethylation in primary human fibroblasts with intact cell cycle checkpoints 6–24 h after irradiation with 2 or 4 Gy. Our aim was to mimic the situation in normal body cells within the first cell cycle after tumor therapy.

## Material and methods

### Cell culture, irradiation, and DNA sample preparation

All experiments were performed with primary human fetal fibroblast strains. Written informed consent was obtained to use anonymized excess materials from prenatal diagnostics for research purposes. With only a few exceptions, male fibroblasts were used to avoid mosaic methylation states of loci subject to X inactivation. Cells were cultured in T25 flasks in Chang Medium with L-glutamine (Irvine Scientific, CA, USA) at 37°C in an incubator with 5% CO_2_ atmosphere. To determine the population doubling time (PDT) 200,000 cells were seeded in a T25 flask. Cells were counted at 8, 24, 48, and 72 h after seeding, using a Fuchs Rosenthal Counting Chamber. The PDT was calculated according to the ATCC Animal Cell Culture Guide: DT = Tln2/ln(Xe/Xb), where T is the incubation time in hours, Xe is the cell number at 72 h and Xb is the cell number at 8 h after seeding.

Medium was changed one day before irradiation and subconfluent cell cultures were selected to reach approximately 90% confluence at the time of harvesting after irradiation. Cells were exposed at room temperature to X-ray doses of 2 or 4 Gy (with a dose rate of 8 Gy/min), using a Siemens Primus L linear accelerator (Siemens, Concord, CA, USA). Cells were harvested at different time points after irradiation and genomic DNA was isolated with the Quick-gDNA MiniPrep kit (Zymo Research, Irvine, CA, USA) according to the manufacturer’s manual. Single cells were isolated using the cell sorting system FACS Aria III (BD Biosciences, San Jose, CA, USA).

### γH2AX foci staining

The number of γH2AX foci was used as a biomarker for DSBs at two different time points after irradiation with 2 and 4 Gy, respectively. Following fixation with 4% paraformaldehyde for 15 min at room temperature, cells were permeabilized with ice cold methanol for 30 min. Cells were then blocked with 20% fetal calf serum in phosphate-buffered saline (PBS). Immunostaining was performed for 1 h at room temperature using the primary rabbit polyclonal anti-γH2AX antibody (pS139, ab11174, Abcam, Cambridge, UK), diluted 1:5000 with PBS, followed by three washing steps in PBS. Secondary staining was performed for 30 min in the dark with goat anti-rabbit Alexa-Fluor 546 (Molecular Probes, Eugene, OR, USA), diluted 1:1000. After washing three times with PBS, preparations were mounted with Vectashield Mounting Medium containing DAPI (Vector Laboratories, Burlingame, USA). Fifty cells per dose and time point were evaluated using a Zeiss Axioskop fluorescence microscope (Zeiss, Oberkochen, Germany).

### Cell vitality assay

The NC-250 Cell Vitality assay (Chemometec, Allerod, Denmark) quantifies decreased cellular levels of reduced gluthation as an early marker of cell death. Cell viability was measured by comparing cells at 6 and 24 h after irradiation with 2 and 4 Gy, respectively, with controls. To this end, cells were loaded on 8-chamber NC-Slides A8^™^ and counted automatically using the NucleoCounter NC-250^™^ system and NucleoView NC-250 software (Chemometec).

### Global DNA methylation/hydroxymethylation measurement

Global 5-mC and 5-hmC levels were quantified using the MethylFlash Methylated DNA and the MethylFlash Hydroxymethylated DNA Quantification kits (Epigentek, Farmingdale, NY, USA). Fluorometric assays were carried out according to the manufacturer’s instructions using 100 ng of input genomic DNA. Measurements were performed in triplicates. Relative fluorescence was determined using a Tecan Reader Infinite 200 at 530_EX_/590_EM_ nm. Absolute amounts and the percentages of 5-mC and 5-hmC were calculated using a standard curve.

### Bisulfite pyrosequencing of repetitive elements

Bisulfite conversion of genomic DNA was performed with the EpiTect Fast DNA Bisulfite kit (Qiagen, Hilden, Germany) according to the manufacturer’s protocol. Isolation and bisulfite conversion of DNA from single cells was done with the EZ DNA Methylation-Direct kit (Zymo Research, Irvine, CA, USA). For single cell analysis, first a multiplex PCR amplifying all three repetitive elements was carried out, followed by separate second-round nested PCRs for ALU, LINE-1, and α-satellite DNA. For cell cultures, only the repeat-specific PCRs were performed using bisulfite-treated DNA without prior multiplex PCRs. The PyroMark Assay Design 2.0 software (Qiagen) was used to design PCR and sequencing primers. Primer sequences and PCR conditions are presented in Supplementary S1 and S2 Tables, respectively. Amplifications were performed with an initial denaturation step at 95°C for 5 min, followed by a repeat-specific number of cycles of 95°C for 30 s, specific annealing temperature for 30 s, and 72°C for 45 s, and a final extension step at 72°C for 5 min. Bisulfite pyrosequencing was done on a PyroMark Q96MD pyrosequencing system (Qiagen) using the PyroMark Gold Q96 CDT reagent kit (Qiagen). The Pyro Q-CpG software (Qiagen) was used for methylation data analysis.

### Methylation array

The Infinium HumanMethylation450K BeadChip (Illumina, San Diego, CA, USA) allows quantification of methylation at more than 485,000 CpG sites covering 99% of RefSeq genes with promoter, first exon, gene body, 5' and 3' untranslated regions (UTRs), and 96% of CGIs. Bisulfite-converted genomic DNAs from 24 (irradiated and control) cultures were whole-genome amplified, enzymatically fragmented, and hybridized to two HumanMethylation BeadChips, according to the manufacturer’s protocol (Illumina). The arrays were scanned with an Illumina iScan.

### Statistical analyses

Statistical analyses were performed with software package R (version 3.2.2). Analysis of variance (ANOVA) was used for between-group comparisons of ELISA and pyrosequencing measurements including treatment group and cell culture as a categorical factor. Post Hoc analysis comparing each treatment against the control has been performed using Dunnett’s many-to-one comparison method as implemented in the multcomp package [27]. For some experiments, paired t-tests were used to compare irradiated cells with controls.

Array data were analyzed using the statistical software package R (version 3.2.2) and the BioConductor platform (version 3.2). Preprocessing was performed using the infrastructure implemented in the minfi [28] and watermelon [29] packages. First, sites with low signal quality (beadcount <3 and detection p-value >0.05) and sites overlapping known SNPs were removed. Furthermore, probes on the sex chromosomes were excluded leaving a total number of 455,264 probes for subsequent analyses. Signal intensity values were normalized using the dasen method as implemented in the watermelon package [29]. To account for potential probe type effects an intra-sample normalization procedure correcting the bias of type-2 probe values (BMIQ) was applied. Differential methylation analysis was performed using the moderated t-test model based on β values as implemented in the limma package [30]. All p values were corrected for multiple testing using the Benjamini-Hochberg method.

## Results

### Cellular radiation effects

Population doubling time of four different primary human fibroblast strains ranged from 21 h to 27 h with an average of 24.1 ± 2.5 h. The growth curves of these representative strains are shown in S1 Fig. DSBs were visualized by γH2AX foci staining in two fibroblast strains at 6 h and 24 h after irradiation with 2 and 4 Gy, respectively (S2 Fig, upper panel). The average number of DSBs was around 8 after 6 h and around four after 24 h, consistent with DSB repair. Cell viability was determined in three independent strains. The percentage of viable cells at 6 h after irradiation was comparable with that of controls. At 24 h, the number of viable cells was slightly lower in cultures irradiated with 2 Gy (-6.9 ± 4.2%) and 4 Gy (-8.2 ± 4.6%), respectively (S2 Fig, lower panel).

### Global 5-mC and 5-hmC levels in irradiated fibroblast cultures

**Fig 1:**
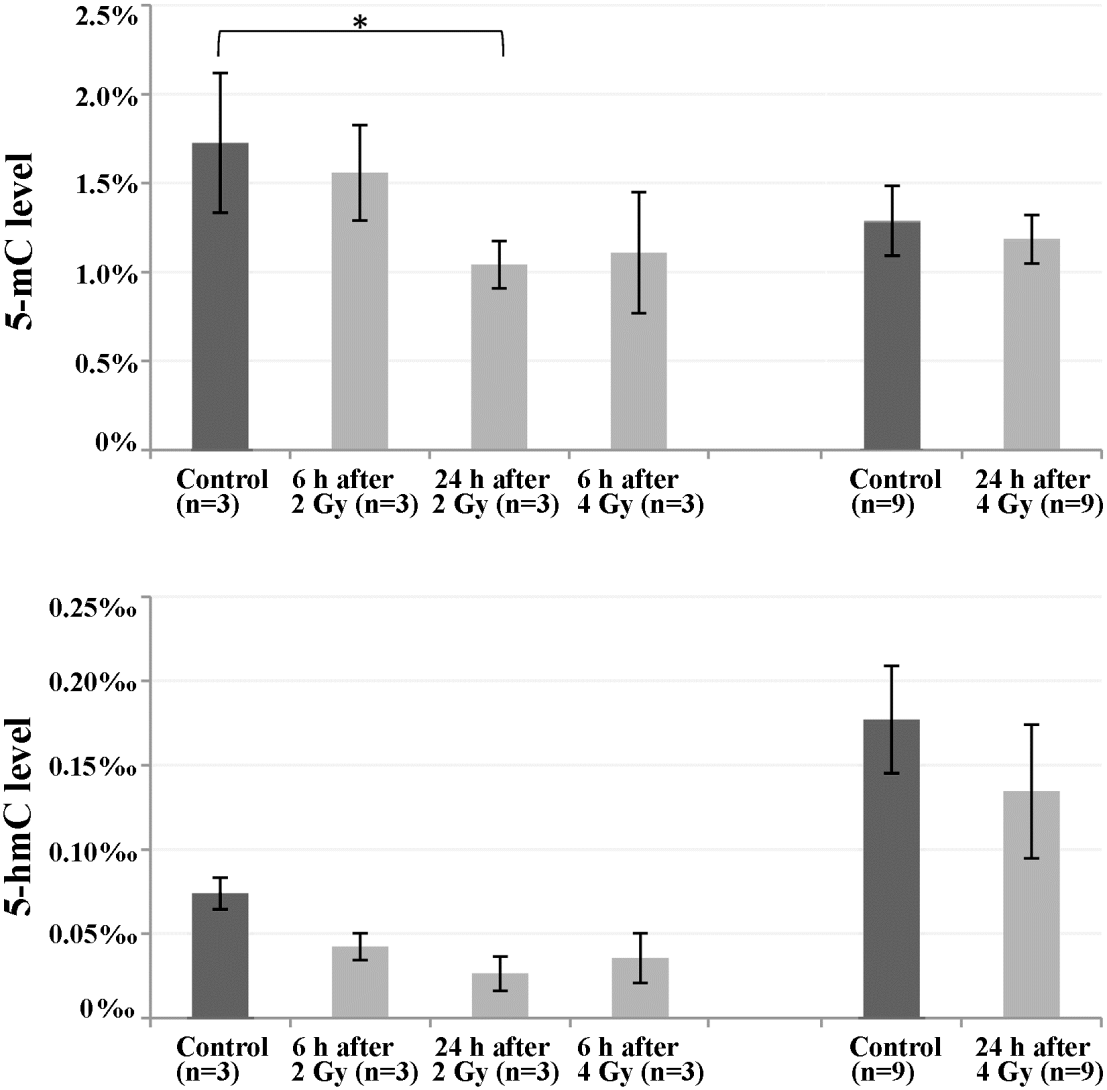
Global DNA methylation and hydroxymethylation in irradiated versus non-irradiated fibroblast cultures. Global 5-mC and 5-hmC levels were measured by ELISA-based assays in primary human fibroblasts at 6 and 24 h after X-ray irradiation with 2 and 4 Gy, respectively. For each time point and dose, the number of analyzed cultures is given in parenthesis. Results are presented as mean (of different cultures) over means (triplicate measurements) ± standard error. Asterisk denotes a significant (p < 0.05) between-group difference.

Several independent approaches were used to analyze global methylation changes in irradiated primary human fibroblasts. First, we employed an ELISA-based fluorometric assay to quantify global DNA methylation and hydroxymethylation at 6 and 24 h after irradiation with 2 and 4 Gy, respectively. The measured 5-mC levels ranged from 1.0% to 1.7% and 5-hmC from 0.03‰ to 0.18‰ (Fig 1). For each dose and time point, global DNA methylation and hydroxymethylation appeared to be slightly reduced in the irradiated cultures. Three independent cultures were measured at 6 and 24 h after 2 Gy as well as at 6 h after 4 Gy and compared with the respective non-irradiated controls. Using an ANOVA (Supplementary S3 Table), the only significant (p = 0.04) between-group difference was observed for global methylation at 24 h after 2 Gy. In an additional experiment 9 irradiated cultures (from 6 different strains) at 24 h after 4 Gy were compared with 9 controls (Fig 1), however no significant (paired t-test) differences were observed.

**Fig 2:**
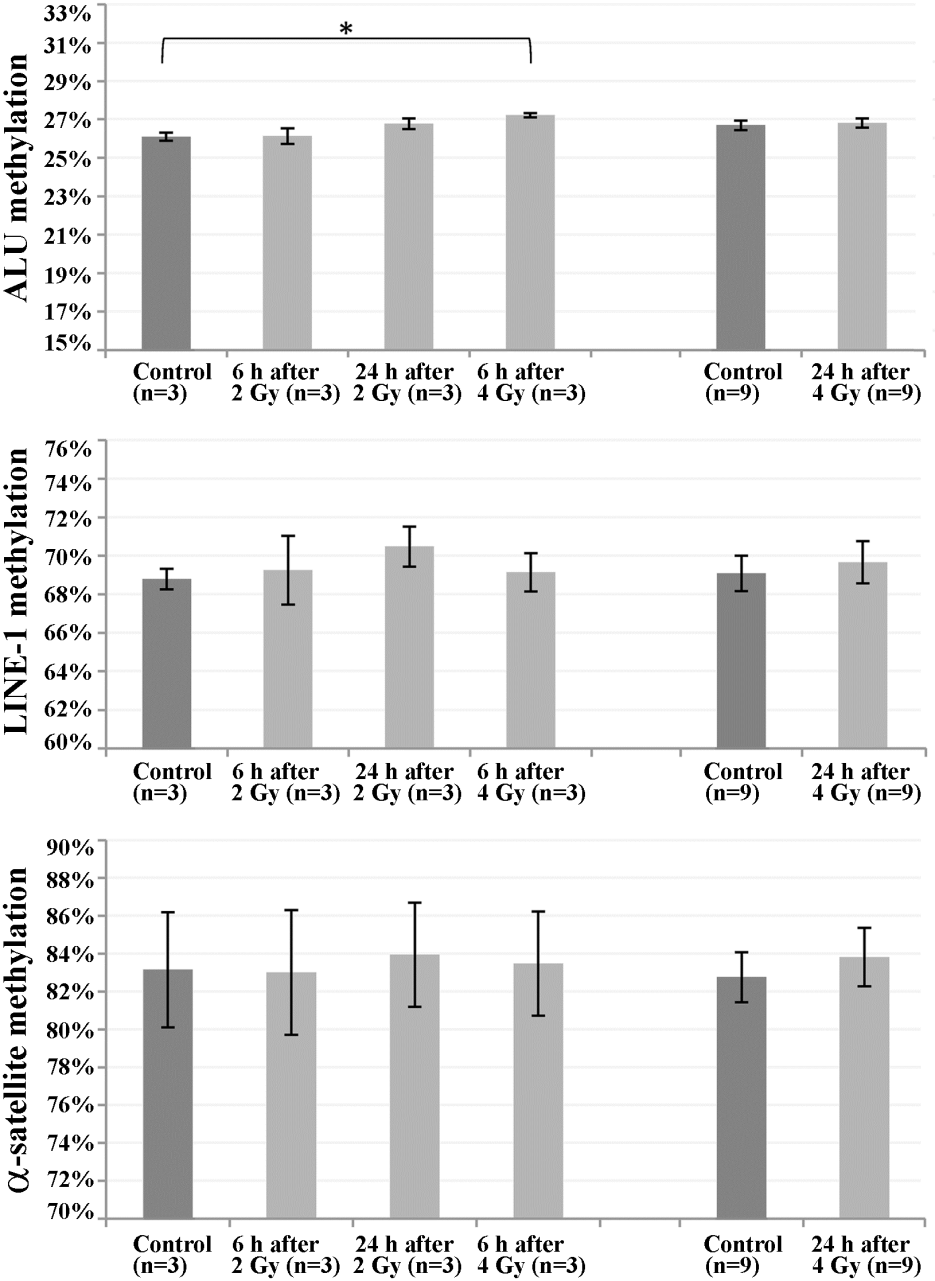
DNA methylation of repetitive elements in irradiated versus non-irradiated fibroblast cultures. Global methylation of interspersed ALU and LINE-1 repeats, and α-satellite DNA was determined by bisulfite pyrosequencing in primary human fibroblasts at 6 and 24 h after irradiation with 2 and 4 Gray, respectively. For each time point and dose, the number of analyzed cultures is given in parenthesis. Results are presented as mean (of different cultures) over means (triplicate measurements) ± standard error. Asterisk denotes a significant (p < 0.05) between-group difference.

The human genome contains approximately 600,000 LINE-1 and more than 1,000,000 ALU interspersed repeats, comprising 17% and 11% of total genomic DNA, respectively [31]. Long arrays (up to several megabases) of α-satellite DNA are present in the centromeric regions of all chromosomes [32]. Since repeats account for more than one-third of methylated CpGs in the genome [12,13], they are frequently used as surrogate markers for detecting genome-wide methylation changes [33]. Bisulfite pyrosequencing with consensus primers was used to determine average methylation levels of ALU, LINE-1, and α-satellite repeats in cultures at 6 and 24 h after irradiation with 2 and 4 Gy, respectively (Fig 2). Consistent with the ELISA results, most between-group comparisons did not yield significant differences. Notable, ALU methylation was significantly (ANOVA; p = 0.01) increased at 6 h after irradiation with 4 Gy (27.2% compared to 26.1% in controls), but not at other time points or lower dose (S3 Table).

Nine irradiated cultures vs. controls were available for 24 h after irradiation with 4 Gy. Therefore, we performed an in-depth analysis of methylation variation. For all 3 repeat families, methylation variation between independent cultures (representing different fetuses and gestational ages) was bigger than the difference between irradiated and non-irradiated cells from the same fibroblast strain (S3 Fig). α-Satellite methylation correlated positively (Spearman Rho 0.785, p = 0.012) with gestational age. Thus, much of the methylation variation between cultures may be caused by developmental methylation trajectories [34,35] and serial passaging (in vitro ageing) of the culture [36,37].

### Methylation of repetitive elements in single cells

**Fig 3:**
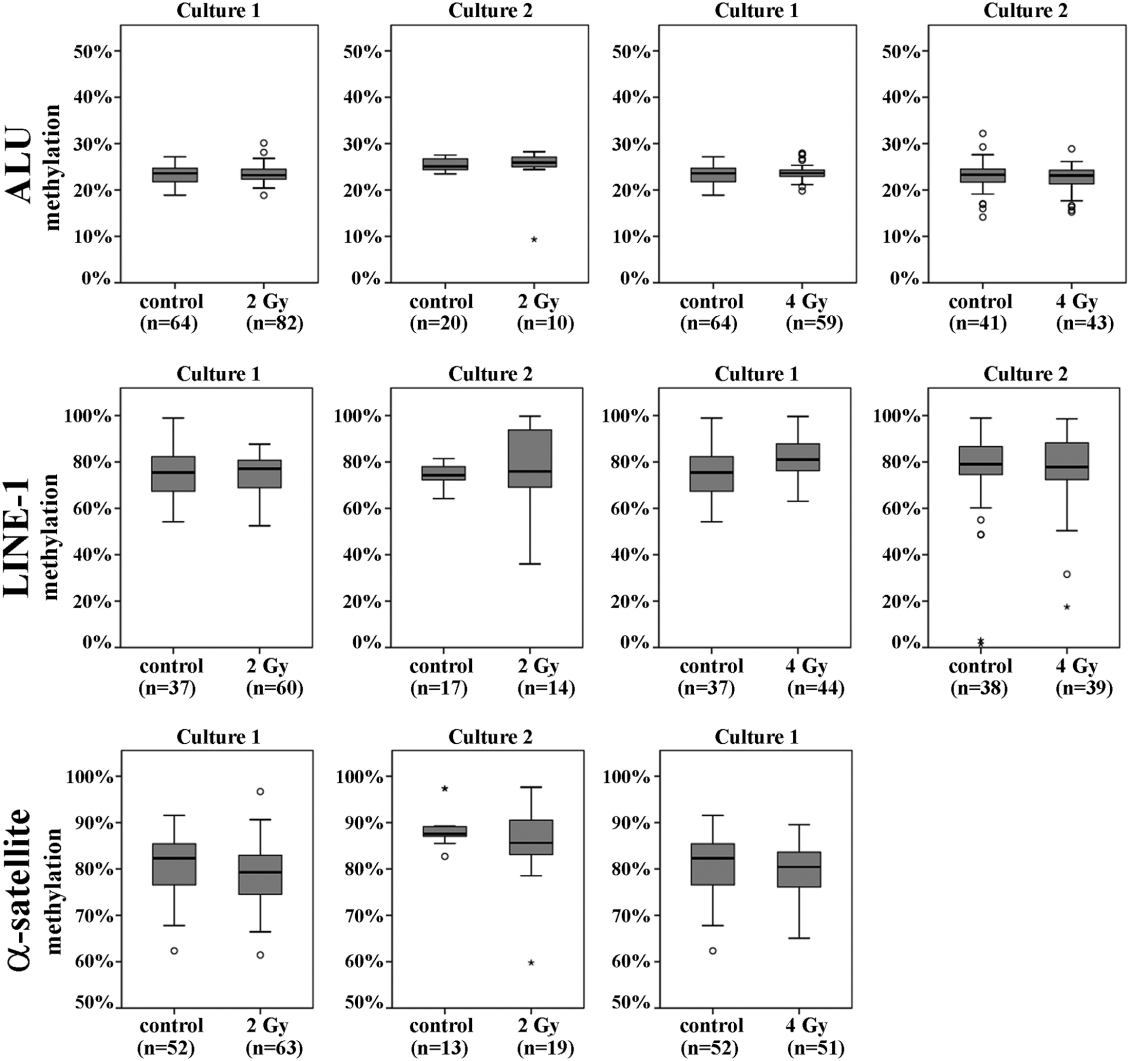
Box plots showing the distribution of repeat DNA methylation in single cells. DNA methylation of interspersed ALU and LINE-1 repeats, and α-satellite DNA was determined by bisulfite pyrosequencing in individual fibroblasts from two independent cultures at 24 h after irradiation with 2 Gy and 4 Gy, respectively. For each culture, time point, and dose, the number of analyzed cells is given in parenthesis. The median is represented by a horizontal line. The bottom of the box indicates the 25^th^ percentile, the top the 75^th^ percentile. Outliers are shown as circles and extreme outliers as stars.

Although global methylation levels remained rather stable in the first 24 h after irradiation, pronounced methylation changes may exist in a few individual cells of a culture, which are then prone to neoplastic transformation. To test this hypothesis, we isolated single cells from two irradiated cultures and their corresponding controls 24 h after irradiation with 2 and 4 Gy, respectively. Methylation of ALU, LINE-1, and α-satellite repeats was measured in the same individual cells, using a multiplex PCR approach. For both radiation doses and both fibroblast cultures, the methylation variation of ALU, LINE-1, and α-satellite DNA was comparable between irradiated and control cells (Fig 3).

### Methylation array analysis

**Fig 4:**
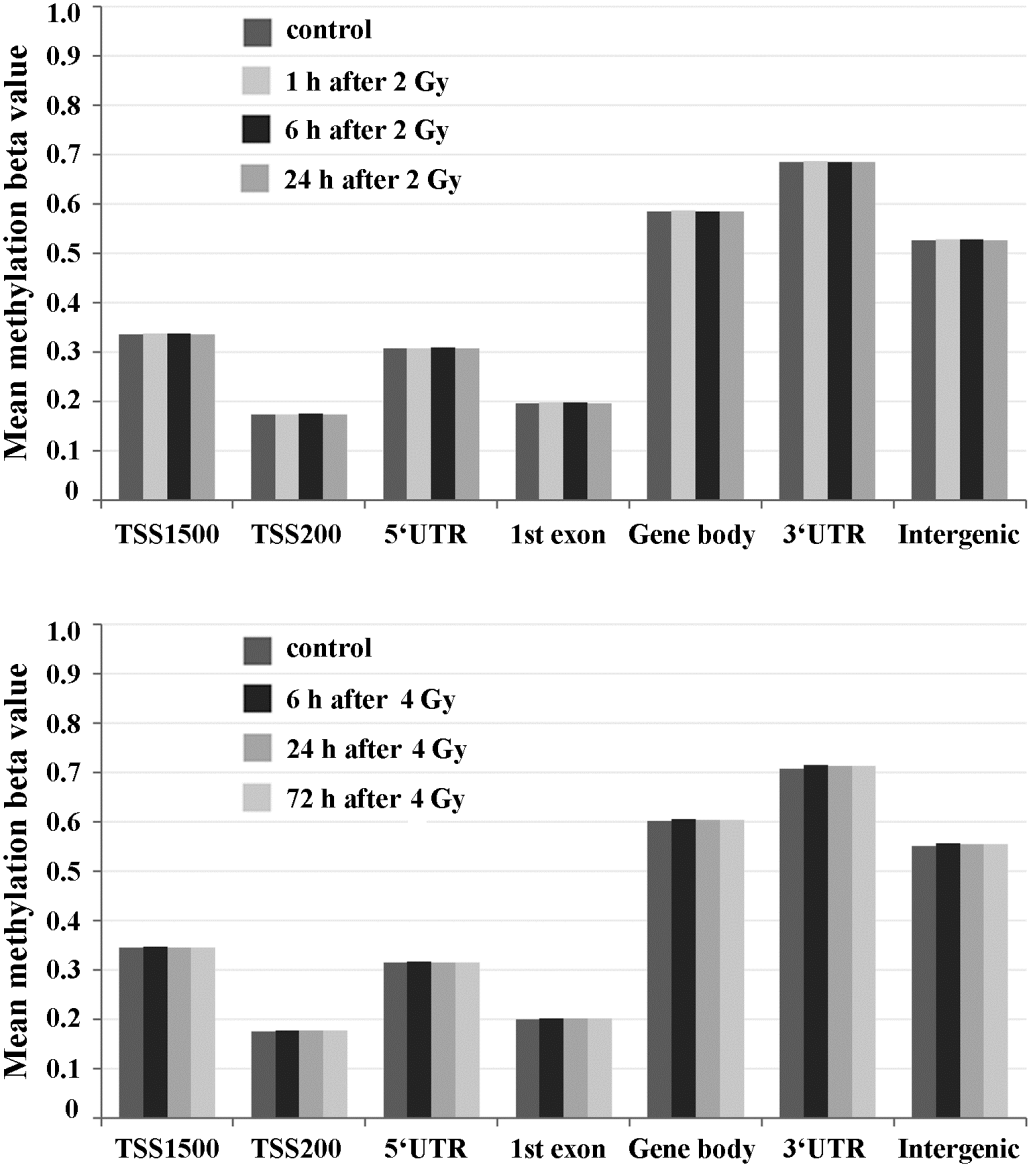
Global DNA methylation in genic and intergenic regions of irradiated versus non-irradiated fibroblast cultures. DNA methylation was assessed with Illumina 450K arrays in primary human fibroblasts at 1–24 h after irradiation with 2 Gy (upper panel) and at 6–72 h after 4 Gy (lower panel). The bars represent the average methylation of all analyzed CpGs that have been annotated to a particular category (promoter, 5' UTR, first exon, gene body, 3' UTR, intergenic). TSS200 is the region from transcription start site (TSS) to -200 bp, TSS1500 from -200 bp to -1,500 bp upstream of TSS. Data are presented as means over means.

To analyze possible radiation effects on non-repeat methylation, we performed methylation array analysis at 1, 6, and 24 h after irradiation with 2 Gy and at 6, 24, and 72 h after irradiation with 4 Gy. For both radiation doses, each assessed time point was represented by one control and three irradiated samples. Most probes on the array are targeted across >99% genes in the genome. Fig 4 shows the average methylation level of all assessed CpGs in promoter regions (defined as DNA segments 1,500 to 200 bp and 200 to 0 bp upstream of the transcription start site), 5' UTRs, first exons, gene bodies, and 3' UTRs. The remaining CpGs are in intergenic regions. An unsupervised cluster analysis of site-specific β values did not separate irradiated and non-irradiated cultures (data not shown). None of the potentially differentially methylated CpG sites reached genome-wide significance. Larger sample sizes are required to detect genes/regions with a direct methylation response to X-ray irradiation.

## Discussion

There are relatively few studies that have analyzed direct epigenetic effects in cells within the first cell cycle after irradiation. Global hypomethylation was observed in the blood of male mice 2 h after irradiation with 0.5 Gy [38] and in thymus tissue of mice 4 h after irradiation with 0.5 Gy [39]. DNA hypomethylation was also seen in human lymphoblastoid TK6 and WTK1 cells within one day after exposure with 2 Gy [5]. Both hypo- and hypermethylated loci were found in breast cancer cells irradiated with 2 and 6 Gy and analyzed at different time points from 1–72 h [3]. In contrast, several studies on normal human cells did not detect significant radiation-induced effects on DNA methylation. Bronchial epithelial cells and fibroblasts were γ-irradiated with different doses and methylation was analyzed seven days after exposure [26]. The same was true for keratinocytes that were repeatedly exposed to UVB and analyzed with a methylated CGI recovery assay combined with microarrays 8 and 18 days after the final dose [40]. Collectively, existing data suggest that radiation-induced effects depend on tissue/cell type, sex, and species [38,41–44]. Moreover, radiation quality, dose, and time after exposure have to be taken into consideration [3,7,8,43].

Many studies were performed on cancer cells or cell lines with compromised DNA repair mechanisms and cell cycle checkpoints. We used primary human fibroblast strains which more closely resemble normal body cells exposed to irradiation during cancer treatment. Methylation was analyzed within the first cell cycle after irradiation to examine early DNA damage response. Average population doubling time of our fibroblast cultures was 24 h. Fractions of 2–4 Gy are commonly applied for radiation therapy of cancer. Exposure to a dose of 2 or 4 Gy perturbs cell cycle progression and activates different cell cycle checkpoints. In our experience (with FACS cell cycle analysis), the percentage of non-cycling cells in exponentially growing fibroblast cultures is approximately tripled at 48 h after irradiation with 1.5 Gy and reaches 40–50%, compared to 15–20% in non-irradiated controls. Primary fibroblast strains with intact cell cycle checkpoint usually arrest in G0/G1 and to a lesser extent in G2 phase, whereas cells with DNA repair defects accumulate in G2 phase [45–48]. Activation of the G1 checkpoint appears to occur slowly and may not yet be completely initiated at 6 h after irradiation, permitting cells with unrepaired DNA damage to enter S phase [49]. γH2AX demonstrated the presence of DNA damage in irradiated cells. The number of foci decreased from approximately 8 per cell at 6 h to four at 24 h, indicating DSB repair after irradiation. Cell viability assays demonstrated that the vast majority of irradiated cells were still viable at 24 h. Compared to control cultures, the percentage of dead cells was only slightly (<10%) increased. Interestingly, there were no dramatic difference between 2- and 4 Gy-irradiated fibroblasts, neither at the epigenetic (DNA methylation) nor at the cellular (DSB induction/repair and cell viability) level.

Using ELISA-based assays, we observed slightly reduced DNA methylation and hydroxymethylation levels in irradiated cells; however significance was only reached for global methylation at one time point and dose. The variation between technical replicates was relatively high, which may be partially due to the low overall levels of 5-mC and in particular 5-hmC in the analyzed cell type. The accuracy of these assays makes it challenging to detect small genome-wide methylation changes [14,16,50] that may occur in normal body cells under various conditions.

Interspersed ALU and LINE-1 repeats have been used previously to assess global DNA methylation changes after radiation exposure. Both low and high linear energy transfer (LET) radiation were reported to induce hypomethylation of ALU and LINE-1 in human-hamster hybrid cells. Interestingly, in one experiment (0.5 Gy of low LET) increased LINE-1 methylation was observed [7]. In another study [51], LINE-1 methylation was increased in peripheral blood cells of occupational radiation-exposed power plant workers. Methylation of satellite 2 DNA which is mainly localized in the large paracentromeric heterochromatin blocks of chromosomes 1 and 16 did not differ between workers and controls. In our study, global methylation of interspersed ALU und LINE-1 repeats and centromeric α-satellite DNA methylation was not greatly altered in irradiated human fibroblasts. The radiation-induced effects on repeat methylation, if any, were smaller than variation between different fetal fibroblast strains. Gestational age [34,35], cell culture conditions, and serial passaging [36,37] may all influence methylation patterns. Although the serial passage numbers of irradiated and control cultures were always identical, numbers differed between experiments. Since the average methylation in a cell population could mask extreme methylation values in individual cells, we performed single cell analysis of ALU, LINE-1, and α-satellite repeats. There was no indication for an increase in methylation variation and number of outliers after irradiation. It is interesting to note that α-satellite DNA methylation positively correlated with gestational age. Apart from its function in gene regulation, DNA methylation also plays a role in chromosome condensation and structure [52]. Increasing methylation of long α-satellite arrays may help to establish/maintain a highly condensed centromeric chromatin structure, ensuring centromere stability and proper chromosome segregation [53].

Illumina 450K arrays were used to analyze genome-wide methylation changes in gene regions. Consistent with a conceptually related study [26] on normal human fibroblasts and bronchial epithelial cells 7 days after irradiation, we did not find global methylation changes in fibroblast 1–72 h after irradiation, neither in genic (promoters, 5'UTRs, first exons, gene bodies, and 3' UTRs), nor in intergenic regions. In contrast, global and gene-specific methylation changes were detected in irradiated breast and colon cancer cells, using the same 450K arrays [3,4]. This argues in favor of the notion that normal and cancer cells differ in their epigenetic response to irradiation. Cells with intact cell cycle checkpoints are arrested after irradiation to allow DNA repair and/or cell death to occur. At least the early phase (within the first cell cycle) of DNA damage response in normal cells does not seem to involve major changes in DNA methylation patterns. Since we studied only two time points, 6 h and 24 h after irradiation, we cannot exclude the formal possibility of dynamic changes in the time period in between. However, this appears to be highly unlikely. In contrast to DNA methylation, which was rather stable within the first cell cycle after irradiation, γH2AX staining demonstrated the dynamic cellular response to DNA damage.

In summary, three independent methods, ELISA-based fluorometric assays, bisulfite pyrosequencing of repetitive elements, and methylation arrays did not provide evidence for gross direct effects of X-ray irradiation on global methylation of primary human fibroblasts. Of course, this does not exclude minor methylation changes at the genome or single gene level as well as changes in histone modifications or microRNA expression. It is also possible that more dramatic methylation changes occur after several replication cycles in surviving cells released from cell cycle arrest. Future experiments with larger sample sizes and different radiation qualities may support the identification of specific genes and pathways which are epigenetically altered by irradiation and may trigger DNA damage response in normal cells.

## Supporting information

S1 Fig Fibroblast growth curves. (DOC)

S2 Fig DNA damage and cell viability after irradiation. (DOC)

S3 Fig Methylation variation of repetitive elements between fibroblast cultures. (DOC)

S1 Table Primers used for methylation analysis by pyrosequencing. (DOC)

S2 Table Multiplex and singleplex PCR conditions for α-satellite, LINE-1, and ALU repeats. (DOC)

S3 Table ANOVA of methylation differences between irradiated cells and controls. (DOC)
