# Supplementary material for: Analysis of global DNA methylation changes in primary human fibroblasts in the early phase following X-ray irradiation
Source: PLoS One. 2017 May 10;12(5):e0177442. doi: 10.1371/journal.pone.0177442 (PMC5425224; doi:10.1371/journal.pone.0177442)
Supplement: S1 Fig — (DOC) [file pone.0177442.s001.doc]

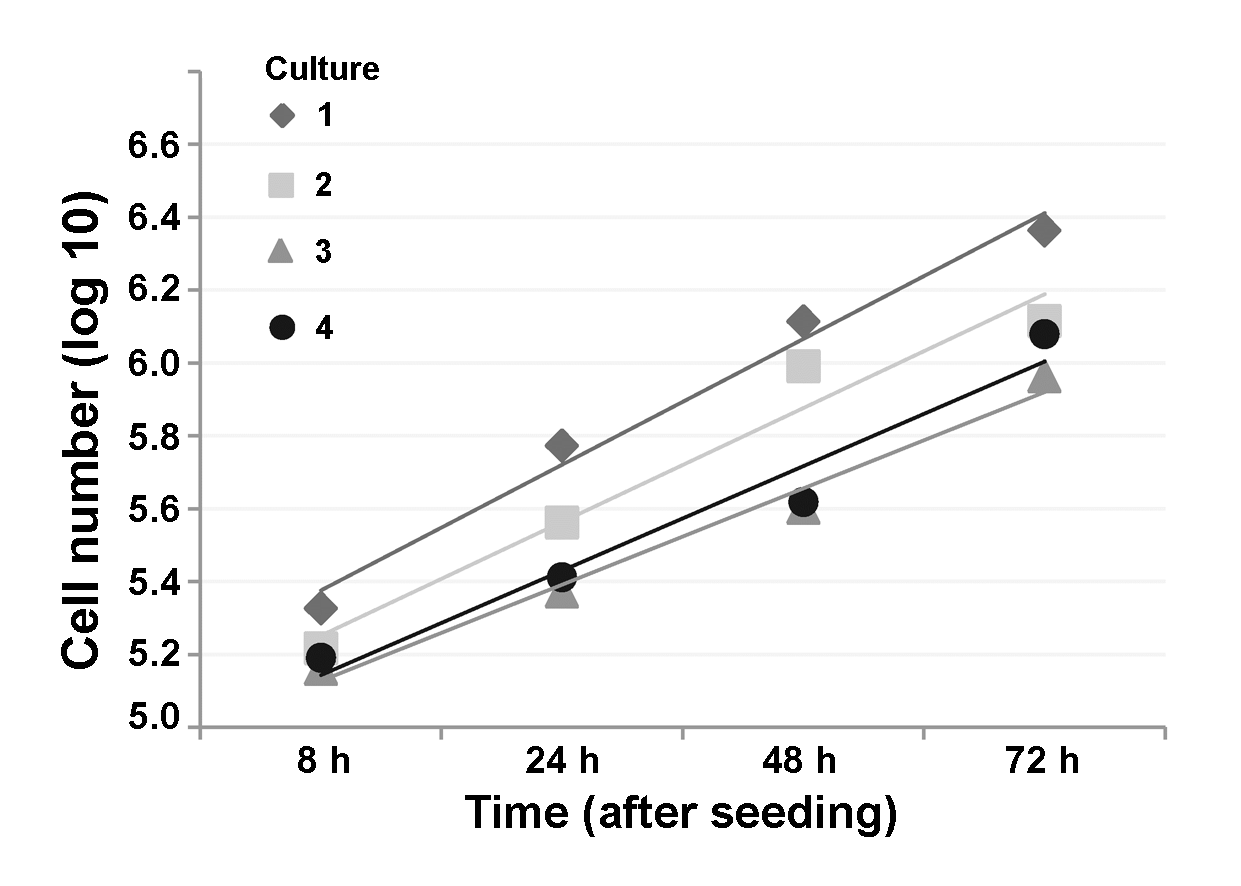


**S1 Figure Fibroblast growth curves.**

The regression lines represent the growth curves of four primary human fibroblast strains (indicated by different symbols). Cell number (logarithmic scale) is plotted against culture time.
