# Supplementary material for: Analysis of global DNA methylation changes in primary human fibroblasts in the early phase following X-ray irradiation
Source: PLoS One. 2017 May 10;12(5):e0177442. doi: 10.1371/journal.pone.0177442 (PMC5425224; doi:10.1371/journal.pone.0177442)
Supplement: S2 Fig — (DOC) [file pone.0177442.s002.doc]

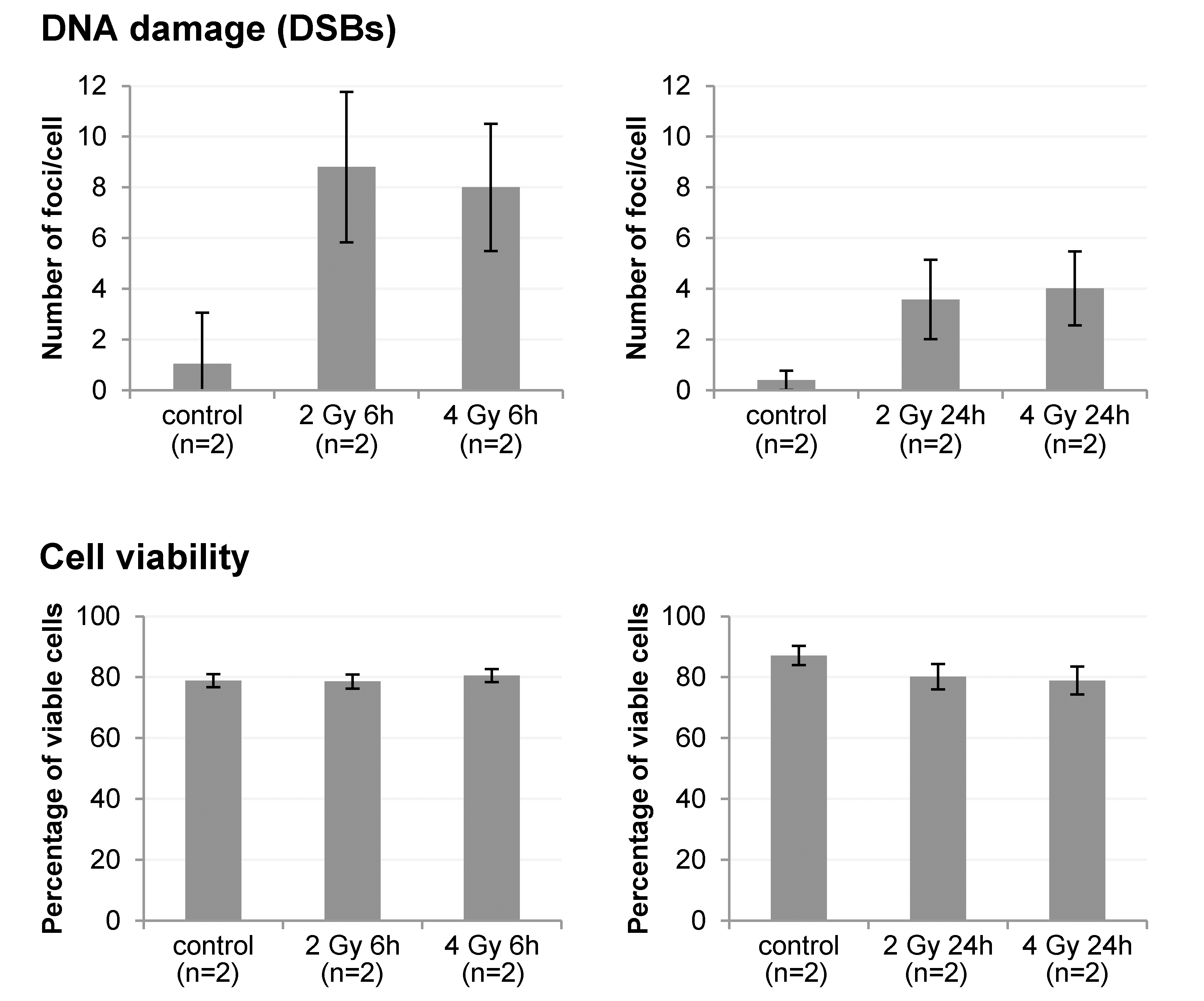


**S2 Figure DNA damage and cell viability after irradiation.**

The upper panel shows the number of DSBs (γ-H2AX foci) per cell at 6 h and 24 h after irradiation with 2 Gy and 4 Gy, compared with non-irradiated controls. Two fibroblast strains and 50 cells per experiment were counted. The bottom panel shows the percentage of viable cells at 6 h and 24 h after 2 Gy and 4 Gy, respectively.
