# Supplementary material for: Analysis of global DNA methylation changes in primary human fibroblasts in the early phase following X-ray irradiation
Source: PLoS One. 2017 May 10;12(5):e0177442. doi: 10.1371/journal.pone.0177442 (PMC5425224; doi:10.1371/journal.pone.0177442)
Supplement: S3 Fig — (DOC) [file pone.0177442.s003.doc]

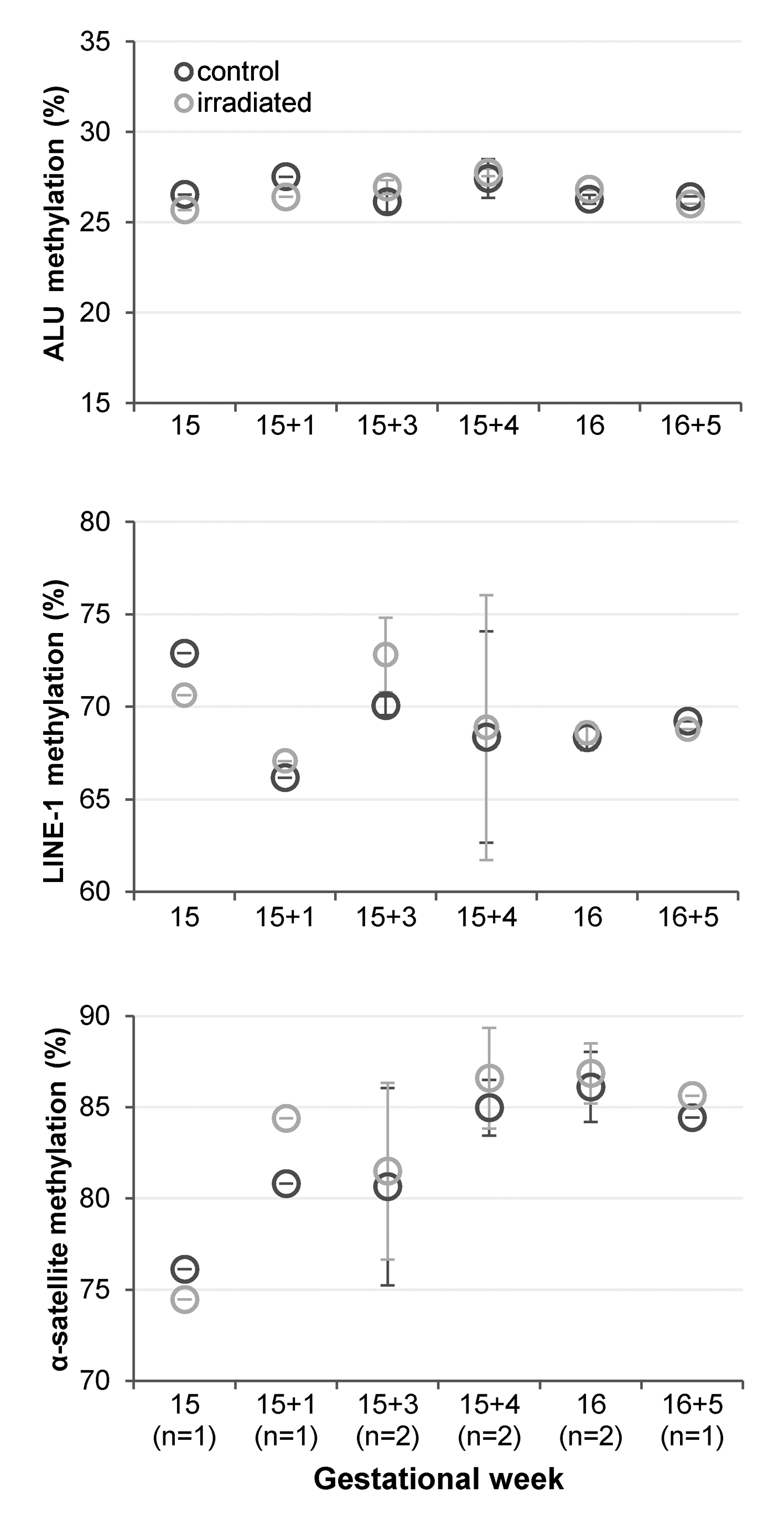


**S3 Figure Methylation variation of repetitive elements between fibroblast cultures.** Global methylation of ALU, LINE-1, and α-satellite repeats was determined by bisulfite pyrosequencing in 6 independent fibroblast strains at 24 h after irradiation with 4 Gray. Methylation variation between irradiated cultures and controls is compared to methylation differences between irradiated and non-irradiated cultures of the same strain. Cultures are arranged according to gestational age. Number of replicates are indicated in parantheses.
