# Supplementary material for: Analysis of global DNA methylation changes in primary human fibroblasts in the early phase following X-ray irradiation
Source: PLoS One. 2017 May 10;12(5):e0177442. doi: 10.1371/journal.pone.0177442 (PMC5425224; doi:10.1371/journal.pone.0177442)
Supplement: S1 Table — (DOC) [file pone.0177442.s004.doc]

| **S1 Table** | | | | | |
| --- | --- | --- | --- | --- | --- |
| Primers used for methylation analysis by pyrosequencing. | | | | | |
| **Assay** | **Primer** | **Sequence (5’-3’)** | **Amplicon length (bp)** | **Repeat**  **Chromosomal localization** | **Number of CpGs** |
| α-satellite | Forward | *TGTAAGTGGATATTTGGATTATTGG | ~160 | Tandem repeats |  |
|  | Outer reverse | TTTCCAAAAAAATCTTCAAAAAAAT |  | All centromeres |  |
|  | Inner reverse | AACAATTTCAAAACTACTCCATCAA |  |  |  |
|  | Sequencing | CTCAAAAATTTCTAAAAATACTTCTC |  |  | 4 |
|  |  |  |  |  |  |
| ALU | Outer forward | TGTAATTTTAGTATTTTGGGAGG | ~150 | Interspersed repeats |  |
|  | Inner forward | GGGACACCGCTGATCGTATA^#^  TTTTTATTAAAAATATAAAAATTAGT |  | All chromosomes |  |
|  | Reverse | CCAAACTAAAATACAATAA |  |  |  |
|  | Universal | *GGGACACCGCTGATCGTATA |  |  |  |
|  | Sequencing | AATAACTAAAATTACAAAC |  |  | 3 |
|  |  |  |  |  |  |
| LINE-1 | Forward | TTTTGAGTTAGGTGTGGGATA | ~245 | Interspersed repeats |  |
|  | Outer reverse | AATTTCTACATTTCCATCTAAAATAC |  | All chromosomes |  |
|  | Inner reverse | *CTCACTAAAAAATACCAAACAA |  |  |  |
|  | Sequencing | GTTAGGTGTGGGATATAGTT |  |  | 4 |
| * 5’-biotinylated primer; ^#^Linker sequence that is recognized by the biotinylated universal primer. | | | | | |
