# Supplementary material for: Analysis of global DNA methylation changes in primary human fibroblasts in the early phase following X-ray irradiation
Source: PLoS One. 2017 May 10;12(5):e0177442. doi: 10.1371/journal.pone.0177442 (PMC5425224; doi:10.1371/journal.pone.0177442)
Supplement: S2 Table — (DOC) [file pone.0177442.s005.doc]

| **S2 Table** | | | | |
| --- | --- | --- | --- | --- |
| Multiplex PCR reaction (for single cells). | | | | |
| **Reagent** | | **Volume for one reaction (µl)** | | |
| 10xPCR buffer with MgCl_2_ | | 2.5 | | |
| dNTPs | | 0.5 | | |
| 10 µM outer forward ALU | | 0.33 | | |
| 10 µM reverse ALU | | 0.33 | | |
| 10 µM forward α-satellite | | 0.33 | | |
| 10 µM outer reverse α-satellite | | 0.33 | | |
| 10 µM forward LINE-1 | | 0.33 | | |
| 10 µM outer reverse LINE-1 | | 0.33 | | |
| Fast Start Taq DNA polymerase | | 0.2 | | |
| Template | | 9 | | |
| ddH_2_O | | 10.82 | | |
| ∑ | | 25 | | |
|  | |  | | |
| Singleplex PCR reaction for α-satellite, LINE1, and ALU repeats, respectively. | | | | |
|  | | **Volume for one reaction (µl)** | | |
| **Reagent** | | **α-satellite or LINE-1** | | **ALU** |
| 10xPCR buffer with MgCl_2_ | | 2.5 | | 2.5 |
| dNTPs | | 0.5 | | 0.5 |
| 10 µM forward primer | | 1.25 | | 1 |
| 10 µM inner reverse primer | | 1.25 | | 1 |
| 10 µM universal primer | | - | | 1 |
| Fast Start Taq DNA polymerase | | 0.2 | | 0.2 |
| Template* | | 1 | | 1 |
| ddH_2_O | | 18.3 | | 17.8 |
| ∑ | | 25 | | 25 |
| *for single cells the multiplex-PCR product was used as template. | | | | |
|  |  | | |  |
|  | **Number of cycles** | | **Annealing temperature** | |
| Multiplex PCR | 35 | | 57°C | |
| ALU | 40 | | 52°C | |
| α-satellite | 40 | | 57°C | |
| LINE-1 | 35 | | 60°C | |
