# Supplementary material for: Analysis of global DNA methylation changes in primary human fibroblasts in the early phase following X-ray irradiation
Source: PLoS One. 2017 May 10;12(5):e0177442. doi: 10.1371/journal.pone.0177442 (PMC5425224; doi:10.1371/journal.pone.0177442)
Supplement: S3 Table — (DOC) [file pone.0177442.s006.doc]

| **S3 Table** | | | | | |
| --- | --- | --- | --- | --- | --- |
| ANOVA of methylation differences between irradiated cells und controls.* | | | | | |
| **Target** | **Group comparison** | **Estimate** | **Standard error** | **Statistic** | **p value** |
| Global methylation 1 | 2 Gy, 6 h vs. control | -0.170 | 0.210 | -0.81 | 0.77 |
| Global methylation 2 | 2 Gy, 24 h vs. control | -0.690 | 0.210 | -3.28 | 0.04 |
| Global methylation 3 | 4 Gy, 6 h vs. control | -0.623 | 0.210 | -2.96 | 0.06 |
| Global hydroxymethylation 1 | 2 Gy, 6 h vs. control | -0.003 | 0.002 | -1.92 | 0.23 |
| Global hydroxymethylation 2 | 2 Gy, 24 h vs. control | -0.005 | 0.002 | -2.86 | 0.07 |
| Global hydroxymethylation 3 | 4 Gy, 6 h vs. control | -0.004 | 0.002 | -2.32 | 0.13 |
| ALU mean 1 | 2 Gy, 6 h vs. control | 0.027 | 0.260 | 0.10 | 1.00 |
| ALU mean 2 | 2 Gy, 24 h vs. control | 0.673 | 0.260 | 2.59 | 0.10 |
| ALU mean 3 | 4 Gy, 6 h vs. control | 1.120 | 0.260 | 4.31 | 0.01 |
| LINE-1 mean 1 | 2 Gy, 6 h vs. control | 0.460 | 0.893 | 0.52 | 0.92 |
| LINE-1 mean 2 | 2 Gy, 24 h vs. control | 1.690 | 0.893 | 1.89 | 0.23 |
| LINE-1 mean 3 | 4 Gy, 6 h vs. control | 0.347 | 0.893 | 0.39 | 0.96 |
| α-satellite mean.1 | 2 Gy, 6 h vs. control | -0.140 | 0.500 | -0.28 | 0.98 |
| α-satellite mean 2 | 2 Gy, 24 h vs. control | 0.797 | 0.500 | 1.59 | 0.34 |
| α-satellite mean.1 | 4 Gy, 6 h vs. control | 0.327 | 0.500 | 0.65 | 0.85 |
| *4 Gy, 24 h was excluded from analysis, because different cell strains were used for this between-group comparison. | | | | | |
